# Elucidating Duramycin’s Bacterial Selectivity and Mode of Action on the Bacterial Cell Envelope

**DOI:** 10.3389/fmicb.2018.00219

**Published:** 2018-02-14

**Authors:** Sahar Hasim, David P. Allison, Berlin Mendez, Abigail T. Farmer, Dale A. Pelletier, Scott T. Retterer, Shawn R. Campagna, Todd B. Reynolds, Mitchel J. Doktycz

**Affiliations:** ^1^Department of Microbiology, University of Tennessee, Knoxville, TN, United States; ^2^Biosciences Division, Oak Ridge National Laboratory, Oak Ridge, TN, United States; ^3^Department of Biochemistry, Cellular and Molecular Biology, University of Tennessee, Knoxville, TN, United States; ^4^Department of Chemistry, University of Tennessee, Knoxville, TN, United States; ^5^Center for Nanophase Materials Sciences, Oak Ridge National Laboratory, Oak Ridge, TN, United States

**Keywords:** duramycin, lipid, phosphatidylethanolamine (PE), peptidoglycan, atomic force microscopy (AFM), lipidomics, cell elasticity, molecular adhesion force

## Abstract

The use of naturally occurring antimicrobial peptides provides a promising route to selectively target pathogenic agents and to shape microbiome structure. Lantibiotics, such as duramycin, are one class of bacterially produced peptidic natural products that can selectively inhibit the growth of other bacteria. However, despite longstanding characterization efforts, the microbial selectivity and mode of action of duramycin are still obscure. We describe here a suite of biological, chemical, and physical characterizations that shed new light on the selective and mechanistic aspects of duramycin activity. Bacterial screening assays have been performed using duramycin and *Populus*-derived bacterial isolates to determine species selectivity. Lipidomic profiles of selected resistant and sensitive strains show that the sensitivity of Gram-positive bacteria depends on the presence of phosphatidylethanolamine (PE) in the cell membrane. Further the surface and interface morphology were studied by high resolution atomic force microscopy and showed a progression of cellular changes in the cell envelope after treatment with duramycin for the susceptible bacterial strains. Together, these molecular and cellular level analyses provide insight into duramycin’s mode of action and a better understanding of its selectivity.

## Introduction

Selective targeting of pathogenic agents is a continuing quest in medical and agricultural applications. One approach in this pursuit is to discover and employ naturally produced antimicrobial compounds. For example, many bacteria manufacture ribosomally synthesized and post-translationally modified peptide (RiPP) natural products (Arnison et al., 2013), a class of peptide-derived compounds that display a narrow spectrum of activity (Lindenfelser et al., 1957). Characterizing the antibiotic capabilities of these molecules expands understanding of the role of antimicrobial natural products in shaping microbiome community structure.

Duramycin and the closely related variant cinnamycin are (methyl)lanthionine-containing RiPPs with known antibiotic activity (Marki et al., 1991). Duramycin is a small, 19 amino acid tetracyclic peptide produced by streptomycetes (Kondo et al., 1964; Kessler et al., 1988; Hayashi et al., 1990; Widdick et al., 2003). This ribosomally-synthesized and post-translationally modified peptide, referred to as a RiPP, contains three thioether bridges formed via two unusual amino acids, lanthionine and methyllanthionine, which stabilize the structure (McAuliffe et al., 2001; Chatterjee et al., 2005; Bierbaum and Sahl, 2009). Enzymatic post-translational installation of three thioether bonds results in a crosslinked structure that is stable to proteolytic activity and creates a stable binding pocket that selectively recognizes the ethanolamine head group of the membrane, phospholipid phosphatidylethanolamine (PE) (Marki et al., 1991; McAuliffe et al., 2001; Hechard and Sahl, 2002). The exquisite phospholipid specificity of duramycin leads to the growth inhibition of some bacteria, fungi and viruses, and thus lanthipeptides such as duramycin may be useful in the treatment of numerous diseases (Gomes et al., 2017).

Although the molecular target of duramycin has been identified, a predictive understanding of its bacterial species selectivity and killing mechanism is less certain. In general, duramycin targets Gram-positive bacteria, although several Gram-negative species can also be affected (Lindenfelser et al., 1957). Certainly, bacterial classification and the considerable differences that can exist in the constituents that encase bacteria at least partially account for duramycin’s selectivity. The cell envelope serves as the first line of defense against both toxic molecules and an unpredictable environment (Silhavy et al., 2010). In Gram-negative bacteria, a thin peptidoglycan cell wall is sandwiched between two phospholipid membranes, with the outer membrane composed predominantly of lipopolysaccharides (Burge et al., 1977; Rietschel et al., 1982; Silhavy et al., 2010). Gram-positive bacteria lack the outer phospholipid bilayer but are surrounded by thicker layers of peptidoglycan (Burge et al., 1977; Silhavy et al., 2010). Peptidoglycan consists of long glycan strands, cross-linked by short peptide bridges (Vollmer and Bertsche, 2008; Vollmer et al., 2008) that protects the cell from damage, resists the outward turgor pressure of the cytoplasm, and confers cellular shape (Burge et al., 1977; Boulbitch et al., 2000). The Gram-positive cell wall is also distinguished by the presence of anionic teichoic acids (Baddiley, 1972; Neuhaus and Baddiley, 2003).

The phospholipid membrane components can also vary significantly between bacterial species and can be asymmetrically distributed across the lipid bilayer (Malanovic and Lohner, 2016a,b). PE, the binding target of duramycin, is the principal zwitterionic phospholipid of microbial membranes and tends to constitute a higher percentage of the membrane in Gram-negative bacteria than in Gram-positive bacteria (Epand and Epand, 2009; Malanovic and Lohner, 2016b). One of the primary roles for PE in bacterial membranes is to disperse negative charges in the overall anionic membrane (Neuhaus and Baddiley, 2003; Epand and Epand, 2009). PE also enables bacterial multidrug transporters to function properly and acts as a chaperone in the assembly of lactose permease (Bogdanov et al., 1996; Gbaguidi et al., 2007). Clearly, molecular capture of PE by duramycin can lead to changes in membrane organization and permeability, disrupting the complex organization and diverse functional roles of the cell membrane and cell wall components (Baddiley, 1972; Leung, 2009). Further, indirect actions of duramycin binding results in inhibition of cell wall synthesis (Marki et al., 1991; Zimmermann et al., 1993). Individually and collectively, these activities can lead to cell death.

The aim of this study was to further elucidate the selective bacteriocidal action of duramycin and its mode of action. For this effort, we examined duramycin activity against the sensitive strain *Bacillus subtilis* 168 as well as a diverse panel of genome sequenced bacteria including nine that were originally isolated from roots of *Populus* trees. Understanding duramycin’s selectivity can aid in its use to selectively reshape *Populus’s* microbiome in defined community experiments (Timm et al., 2016). A range in sensitivity to duramycin is observed that correlates closely with chemical and physical phenotypes obtained from high resolution imaging, single molecule recognition studies and lipidomic analyses. The results of these investigations reveal new details regarding duramycin’s selectivity and interaction with the cell surface.

## Materials and Methods

### Bacterial Strains and Chemical Reagents

The nine bacterial strains originally collected from the roots of *Populus deltoides* and *Populus trichocarpa* (Gottel et al., 2011; Brown et al., 2012; Shakya et al., 2013) include: *Chryseobacterium CF314, Herbaspirillum YR522, Variovorax CF313, Bacillus BC15, Paenibacillus BC26, Pseudomonas GM17, Arthrobacter CF158, Rahnella OV744*, and *Sphingobium AP49.* Also included was *B. subtilis 168* (*ATCC 6633*) (Kunst et al., 1997). The strains are summarized in **Table 1**. Duramycin from *Streptoverticillium cinnamoneus* (≥90.0%; MW 2013.28) was obtained from Sigma–Aldrich (St. Louis, MO, United States). Duramycin-LC- Fluorescein (D-1001) was purchased from Molecular Targeting Technologies (West Chester, PA, United States). PE was purchased from Avanti Polar Lipids (Alabaster, AL, United States).

**Table 1:** Examined bacteria and their duramycin sensitivity.

Strains	Gram staining	Accession No.	MIC (μM)
*Bacillus subtilis 168*	Gram positive	NZ_CP010052	2
*Bacillus BC15*	Gram positive	NZ_FRBJ01000017	3.75
*Herbspifillum YR522*	Gram negative	NZ_AKJA00000000	17.5
*Paenibacillus BC26*	Gram positive	FPAD00000000	17.5
*Variovorax CF313*	Gram negative	NZ_AKIW00000000	20
*Chryseobacterium CF314*	Gram negative	NZ_AKJY00000000	21
*Sphingobium AP49*	Gram negative	NZ_AJVL00000000	32.5
*Rahnella OV744*	Gram negative	NZ_JUHM00000000	43
*Pseudomonas GM17*	Gram negative	NZ_AKJU00000000	100
*Arthrobacter CF158*	Gram positive	FNNR00000000	200


Duramycin conditioned *Arthrobacter CF158, Pseudomonas GM17, Bacillus BC15*, and *B. subtilis 168* were grown overnight in R2A media with 1 μm duramycin at 30°C with shaking. Cells from each of the strains were inoculated again into fresh R2A media containing 1 μm duramycin with shaking for 18–24 h. Centrifugation at 3,381 RCF for 5 min was used to separate pellets of growing cells from the supernatant. The cell pellets were stored at -80°C for further study.

Other chemicals used in the study were, aminopropyltriethoxy silane (APTES), and triethylamine, 4-morpholineethanesulfonic acid hemisodium salt (MES), from Sigma–Aldrich (St. Louis, MO, United States). From Thermo Fisher (Rockford, IL, United States), we obtained ethyl-3-(3-diethylaminopropyl) carbodiimide HCL and sulfo NHS. Gelatin, used for mounting and imaging the bacteria by AFM was porcine gelatin #2500 from Sigma–Aldrich (Doktycz et al., 2003; Hasim et al., 2017). R2A broth media was obtained from Sigma–Aldrich (St. Louis, MO, United States) and for R2A agar plates we added 20 g of bacteriological agar to 1 L of R2A media.

### Selectivity and Minimum Inhibitory Concentrations

Overnight cultures of the bacterial strains were diluted 1:10 in R2A media (OD∼0.1) and 100 μL of each of the cultures was pipetted onto R2A agar plates. For each of the strains a sterile cotton swab was dipped into the diluted culture and used to streak the plate three times. A number of duramycin stock solutions, with concentrations ranging from 200 to 0.025 μM, were filter sterilized (0.2 μm), and 2 μL of each diluted duramycin concentration was spotted directly onto small circular filter paper pieces placed in the center of each lawn. Plated bacteria were left to grow overnight at room temperature. Inhibition, evidenced by the creation of clearance zones around the circular filter paper, was monitored over the next 2 days. Inhibition tests were performed in triplicate. We also used a broth microdilution technique in which 100 μL of R2A media, with increasing concentrations of duramycin ranging from 200 to 0.025 μM, was dispensed into the wells of microwell plates with a known number of bacteria (10^4^ cells/mL) and OD_600_ measurements were then recorded using an EnSpire multimode plate reader (PerkinElmer) to determine results after overnight growth.

### Fluorescence Microscopy to Visualize Duramycin Binding to PE on the Surface

Strains were grown overnight in R2A at 30°C. Cells from each of the strains were then washed with phosphate-buffered saline (PBS) and diluted to an OD_600_ of 0.1. Cells were mixed with 50 ng Duramycin-LC- Fluorescein for 40 min at 30°C followed by 3X centrifugation (3,381RCF) followed by several rinsing steps in PBS. In the final centrifugation, 100 μL of PBS was added to the pellet, mixed, placed on a glass slide and observed by confocal microscopy using a Zeiss LSM 710.

### Lipid Isolation for Mass Spectrometry Analysis

Each of the 10 bacterial strains tested was grown overnight, with shaking at 30°C in 3 mL of R2A. Lipid isolations were adapted from the protocol of Singh et al. (2010). Briefly, cells were diluted to 0.4 OD_600_ in 50 mL of R2A media followed by shaking at 30°C for 6 h. OD_600_ values were recorded for normalization and pellets were suspended in 1 mL of PBS containing 100 μL of a pre-warmed stock solution of 100 mg/mL lysozyme. Cells were incubated for 15 min at 37°C and lysed using 0.5 g of glass beads (Sigma–Aldrich, St. Louis, MO, United States) and a mini beadbeater (Biospec products). 1.5 mL methanol and 3 mL chloroform were added to the lysed cells and centrifuged for 5 min at 1150 RCF. The middle organic layer was transferred to a second glass tube, leaving behind the beads and cell debris. The organic layer was then dried under nitrogen gas and stored at -20°C. Immediately before mass spectrometric analysis, the lipid extracts were resuspended in 300 μL of 9:1 methanol: chloroform (v/v).

### Mass Spectrometry Lipidomics

Lipid extractions were carried out for two of the sensitive strain (*Bacillus BC15, B. subtilis 168*) and two of the resistant strains (*Arthrobacter CF158, Pseudomonas GM17*). Lipidomics methods, including extraction and mass spectrometric detection, were adapted from Cassilly et al. (2017) with the additional use of external standards from each phospholipid class to verify retention times. The relative abundance of each phospholipid species was used for comparisons since internal standards were not used in this study. A Kinetex HILIC column (150 mm × 2.1 mm, 2.6 μm) (Phenomenex, Torrance, CA, United States) connected to an Ultimate 3000 autosampler and UHPLC pump was used to introduce the analytes into an Exactive benchtop Orbitrap mass spectrometer (Thermo Fisher Scientific, San Jose, CA, United States) equipped with an electrospray ionization (ESI) probe. The separation used 10 mM aqueous ammonium formate pH 3 (mobile phase A) and 10 mM ammonium formate, pH 3 in acetonitrile:water (93:7, v/v) at a flow rate of 0.2 mL/min with the following gradient: *t* = 0 min, 0% A, 100% B; *t* = 1 min, 0% A, 100% B; *t* = 15 min, 19% A, 81% B; *t* = 15.1 min, 52% A, 48% B; *t* = 25 min, 52% A, 48% B; *t* = 25.1 min, 0% A, 100% B; *t* = 35 min, 0% A, 100% B (end of analysis). The column temperature was 25°C and the autosampler temperature was 4°C. The MS spray voltage was 4 kV, and the heated capillary temperature was 350°C. The sheath gas flow rate was 25 units, and the auxiliary gas flow was 10 units. Ions were collected for every sample in both positive and negative mode using these conditions. External mass calibration for the instrument was performed using the manufacturer’s calibration mixture and protocol every 2 days. The resolution of the MS was 140,000 with a scan range of 100–1500 *m/z* was used in the analyses. Lipidomics data were processed using the Maven software (Melamud et al., 2010), and lipids were identified using a combination of exact *m/z* and retention times. Lipid standards (Avanti Polar Lipids, Alabaster, AL, United States) from each phospholipid class were run to verify retention times. Three separate biological replicates and two technical replicates were carried out for each strain, and the data were normalized by dry weight.

### AFM Imaging and Elasticity Measurements Before and After Treatment with Duramycin

For imaging, overnight cultures of bacteria were centrifuged at 3,381 RCF for 10 min. The pellet suspended in 1 mL of R2A media and 300 μL were transferred into new tubes containing 3 mL of R2A media. Strains were treated with 1 μM of duramycin and incubated at 30°C with shaking. After treatment for 1–3 h, 1 mL was withdrawn from the bacterial samples, spun for 5 min at 3,381 RCF, the supernatant was discarded, and the pellets resuspended in 500 μL of sodium acetate buffer (Formosa et al., 2013). 100 μL droplets of each of the bacterial treated samples were then placed on gelatin covered mica for 5 min for immobilization (Doktycz et al., 2003; Hasim et al., 2017). Each sample was then imaged in water using a 5500 PicoPlus AFM operating with the 1.20.2 operating system (Keysight Technologies, Inc., Santa Rosa, CA, United States). The instrument was operated in contact mode using MLCT probes with spring constants of 0.03 N/m with a tip radius of 10 nm and Poisson ratio of 0.5. For both imaging and elasticity measurements the applied force was kept at 3–5 nN.

For elasticity measurements, cells were mounted as for imaging and force-volume maps collected. A 0.5 μm square scan area consisting of 16 × 16 points, positioned on the top of the cell was measured. Each point was an average of three force curves and an average of 10 cells for each of the strains was recorded. The Young’s modulus was calculated using the Keysight software.

### Single-Molecule Force Study

For cantilever tip functionalization, the protocol described in Hinterdorfer et al. (1996) and Hasim et al. (2017), and based on information supplied by Dr. Hermann J. Gruber, Johannes Kepler University, Linz, Austria was used. Briefly, after washing in chloroform and drying AFM cantilevers, a 5 L desiccator was flushed with argon gas and cantilevers were placed on the platform. 30 μL of APTES and 10 μL of trimethylamine placed in separate microcentrifuge tube lids on the platform and the desiccator was purged with argon gas for 2 min and the cantilevers were incubated for 2 h. For further functionalization, 1 mg of NHS-PEG (25)-COOH was diluted in 0.5 mL of chloroform and mixed with 30 μL of trimethylamine and incubated for 2 h in the desiccator. After washing in chloroform the cantilevers in droplets of, 1.1 mg of Sulfo-NHS and 0.4 mg of EDC dissolved in 1 mL of 0.1 M MES buffer at pH 6.1, placed on parafilm for 15 min followed by rinsing four times in PBS. Droplets of the newly diluted duramycin (1 × 10^13^ molecules/mL) in PBS were placed on parafilm and the cantilevers were submerged in this solution for 2 h rinsed and stored in PBS. The adhesion-force between the duramycin functionalized tips (force constant 0.03 N/m) and the bacterial cell surface was measured using a 16 × 16 grid of points in a 0.5 μm × 0.5 μm square area. This information was then processed by Keysight software to attain the binding force and frequency.

### Statistical Analysis

Graphs were prepared using OriginPro8 and GraphPad Prism version 7. Unpaired *t*-tests were used to determine significance between results.

## Results and Discussion

### Identifying Bacteria Resistant to Duramycin via Measurements of the Minimum Inhibitory Concentration and Growth Rate

To evaluate duramycin selectivity, and microbial sensitivity to duramycin, 10 bacterial strains were initially evaluated (**Table 1**). The minimum inhibitory concentration (MIC) of duramycin in sterile R2A broth was determined and a range of activity was observed. A value of 50 μM was used as a MIC cutoff for resistant strains. Most of the strains were sensitive to duramycin and displayed MIC values between 2 and 43 μM (**Table 1**). Two strains were largely resistant to duramycin (MIC values of 100 to 200 μM) (**Table 1**). Duramycin sensitivity on R2A agar was also determined by noting the lowest concentration of duramycin that caused a clearance zone on the bacterial lawn (Supplementary Figure S1) and indicated a ranking in sensitivity consistent with those found in solution. In general, however, the sensitivity values determined using agar plate cultures were higher than those determined in solution. This is likely due to differences in antibiotic diffusion or uneven application on the filter disk (Daley et al., 2016).

**FIGURE 1:**
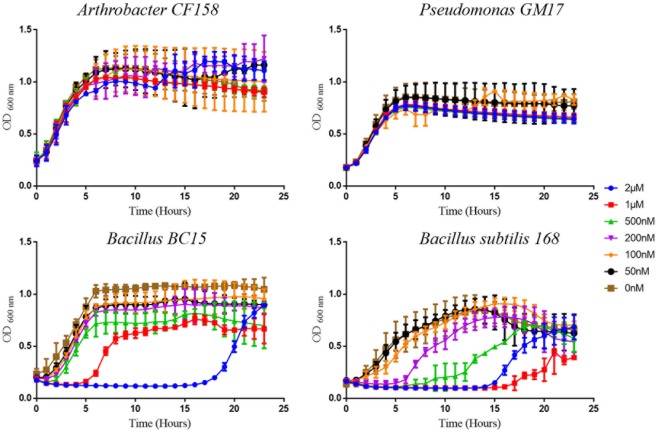
Growth curves of bacterial strains treated with duramycin. The growth curves of *Arthrobacter CF158* and *Pseudomonas GM17*, the two resistant bacterial strains and *Bacillus subtilis 168* and *Bacillus BC15*, the two sensitive strains, in the presence of various concentrations of duramycin in R2A growth media are shown. In the absence of duramycin, no major differences in growth rates are observed between the strains. Further, for the resistant strains, *Arthrobacter CF158* and *Pseudomonas GM17*, duramycin concentrations up to 2 μM do not affect bacterial growth. However, growth of *Bacillus BC15* and *B. subtilis 168* is inhibited at 500 and 200 nM duramycin, respectively. Figures show the average data of three biological measurements, and standard deviations were all less than 3%.

Among the 10 strains, the two most sensitive (*Bacillus BC15*, and *B. subtilis 168*) and the two least sensitive (*Arthrobacter CF158, Pseudomonas GM17)* to duramycin were selected for further study. Growth curves in the presence and absence of different concentrations of duramycin were measured (**Figure 1**). In the absence of duramycin, all strains display similar growth rates. In the presence of increasing concentrations of duramycin, the growth rates of the resistant strains (*Arthrobacter CF158* and *Pseudomonas GM17)* appear unaffected while the sensitive strains (*Bacillus BC15* and *B. subtilis 168*) show increasing lag times preceding growth.

To evaluate the origin of this delayed growth, the stability of duramycin in growth media was assessed. For this test, 1 μM duramycin was incubated overnight at 30°C in media and then used in growth assays. No change in growth profile was observed, indicating that the cellular growth rate is not related to the half-life of duramycin in the media (data not shown). As a second test, cells were plated after 3 and 6 h of growth in the presence and absence of duramycin to determine if duramycin is bactericidal or bacteriostatic. As shown in Supplementary Table S1, a similar number of colonies were obtained under both conditions, indicating that the cells are growth inhibited, but not killed, when exposed to duramycin. To ascertain that the observed cellular growth is not due to contaminating cells, DNA was extracted from cells grown overnight in the presence of duramycin and 16S rRNA sequencing performed. These results indicate that the cells at the beginning of inoculation are the same as those after 24 h of growth (data not shown).

**FIGURE 2:**
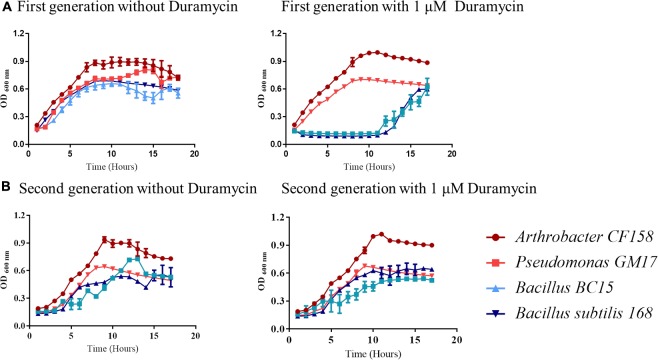
Growth curve comparisons for first and second-generation cells exposed to duramycin. **(A)** First-generation bacteria grown without and with 1 μM duramycin. **(B)** Second-generation bacteria grown without and with 1 μM duramycin. Growth of the second-generation *Bacillus BC15* and *B. subtilis 168* strains, in presence of 1 μM duramycin, are improved relative to the first-generation cells. Figures show the average data of three biological measurements, and standard deviations were all less than 3%.

Collectively, the growth results and verifications indicate that duramycin does not kill the sensitive bacterial strains during initial exposure to duramycin but instead leads to a retardation of growth that result in development of resistance (**Figures 1, 2**). The bacteria are likely in a static state and adaptation to duramycin may result in part by mutation or gene regulation. To assess this possible adaptation, cells, inoculated from overnight cultures that were exposed to 1 μM duramycin, were used to inoculate fresh R2A media with different concentrations of duramycin. As shown in **Figure 2**, these “conditioned” second-generation cells show tolerance to duramycin. The change or mutation appears to be stable as regrowth of isolates of the conditioned, second-generation cells, after storage at -80°C, produce the same results. Further detailed investigations will be needed to identify the molecular underpinnings for this acquired resistance.

### Bacterial Membrane Characterization

To assess for binding of duramycin to the different bacterial strains, a fluorescein labeled duramycin probe was employed. As shown in Supplementary Figure S2, a significant amount of duramycin binding to the sensitive, Gram-positive *Bacillus BC15* and *B. subtilis 168* cells is observed. No binding is apparent to the resistant, Gram-positive *Arthrobacter CF158.* Notably, binding is observed to resistant *Pseudomonas GM17* cells. Duramycin is known to have a high binding affinity to the membrane lipid PE, which is an especially large component of the outer membrane of Gram-negative bacteria (Marki et al., 1991; Zimmermann et al., 1993; Epand and Epand, 2009; Malanovic and Lohner, 2016a). When the duramycin-conditioned, second-generation strains were treated with duramycin-LC-fluorescein, only *Pseudomonas GM17* exhibited binding. These observations imply that the resistant *Arthrobacter CF158* and the resistant, second-generation *Bacillus BC15* and *B. subtilis 168* cells, have low levels of PE in their membrane.

**FIGURE 3:**
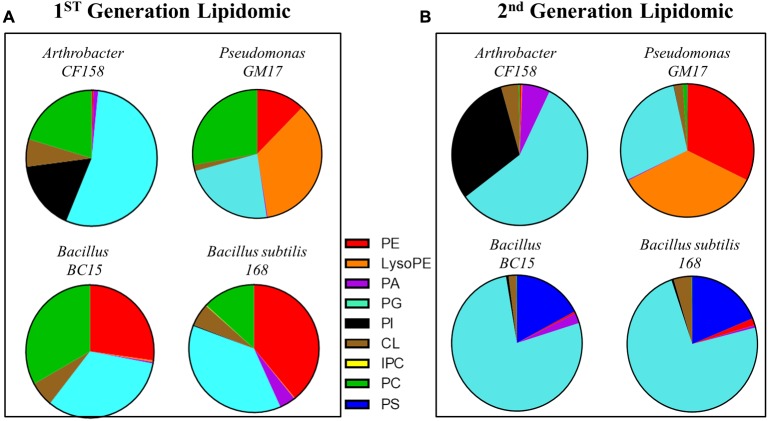
Lipodomic analyses of duramycin sensitive and resistant strains. **(A)** The phospholipid species profiles are shown for the indicated duramycin resistant (top) and sensitive cells (bottom). The sensitive, Gram positive strains contain high levels of PE. **(B)** Shows the phospholipid species profiles for the same cells that have been conditioned with duramycin. All cell strains have changed their phospholipid profiles. PA, phosphatidic acid; PC, phosphatidylcholine; PG, phosphatidylglycerol; PE, phosphatidylethanolamine; PS, phosphatidylserine; PI, phosphatidylinositol; CL, cardiolipin.

To further define the phospholipid components of the bacterial membranes, lipidomic analyses were performed on the four test strains and their duramycin conditioned second-generation counterparts. **Figure 3** displays the relative proportions of the major phospholipids, phosphatidic acid (PA), phosphatidylglycerol (PG), cardiolipin (CL), phosphatidylserine (PS), phosphatidylcholine (PC), and PE as determined by UHPLC-ESI-MS. A summary of the lipid components can be found in the Supplementary Table S2 (first-generation) and Supplementary Table S3 (second-generation). Consistent with the duramycin-LC-fluorescein staining results, the duramycin sensitive *Bacillus BC15* and *B. subtilis 168* strains and the Gram-negative *Pseudomonas GM17* have a relatively high content of PE compared to the duramycin resistant *Arthrobacter CF158*. For the duramycin-conditioned, second-generation cells there is a notable loss of PE and PC and a gain in PG for the previously duramycin sensitive *Bacillus BC15* and *B. subtilis 168* strains. The nominally resistant strains *Arthrobacter CF158* and *Pseudomonas GM17* also show a clear loss of PC.

These changes in phospholipid composition can be reconciled by considering their biosynthetic pathways (Supplementary Figure S3). In bacterial cells, PE and PC are the downstream products of the precursor PS. In order to balance membrane integrity and resist duramycin binding, the previously sensitive Gram-positive cells may respond by mutating genes involved in decarboxylating PS to PE, thereby accumulating the precursors PS and CDP-DAG and decreasing PE. This accumulation of CDP-DAG may then lead to increased PG formation, as PG is also derived from CDP-DAG along with PS. The nominally resistant Gram-positive *Arthrobacter CF158*, which produces little to no PE in the first-generation, appears to reduce flux through the PS/PE/PC pathway, and increases flux through the alternative PI pathway (Supplementary Figure S3). Again, CDP-DAG is a precursor for PI synthesis along with PS, so decreased synthesis of PS and resulting downstream products could cause increased synthesis of PI due to CDP-DAG build-up. The location (inner or outer membrane) of this increased PE content cannot be determined by these lipidomic analyses. The buildup of this duramycin target may be on the inner membrane of the bacterium where its exposure to duramycin would be reduced.

These biochemical characterizations of the bacterial membrane highlight the correlation of duramycin sensitivity and phospholipid composition. For the Gram-positive bacteria, there is a close relationship between duramycin activity and the PE composition of the membrane. In all cases, exposure of bacterial cells to duramycin leads to changes in lipid composition and lipid metabolic flux away from the PS-PE-PC pathway. This could be due to changes to, or regulation of, the phosphatidylserine decarboxylases (PSDs) used for converting PS to PE or to regulation at the upstream pathway that diverts metabolism toward the synthesis of alternative phospholipids.

### Physical Characterization by Atomic Force Microscopy

**FIGURE 4:**
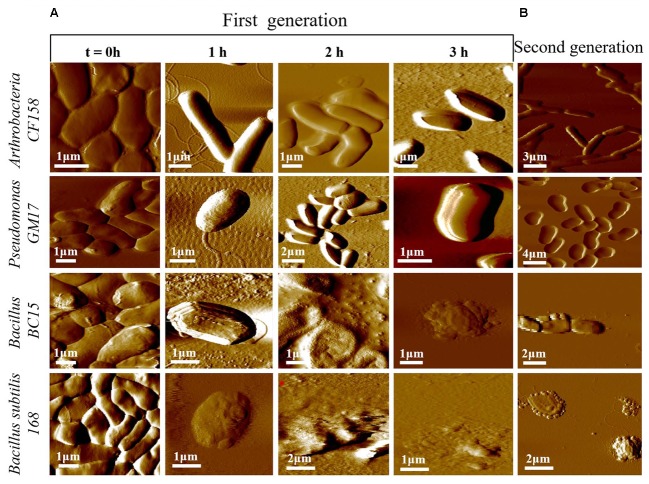
Time course AFM images of duramycin resistant and sensitive cells. **(A)** The duramycin resistant strains, *Arthrobacter CF158* and *Pseudomonas GM17*, show no change in surface ultrastructure and morphology with increasing duramycin exposure time while the duramycin sensitive strains, *Bacillus BC15* and *B. subtilis 168*, show progressive cell destruction. Images were collected at time zero, before duramycin treatment, and after 1–3 h of duramycin treatment. **(B)** The morphology changes between first-generation and duramycin conditioned, second-generation cells, without duramycin treatment, can be compared. The previously sensitive *Bacillus BC15* and *B. subtilis 168* strains show altered morphology, especially the *B. subtilis 168* strain.

To better understand the functional consequences of duramycin activity and its species selectivity, high resolution imaging and physical characterization by AFM was performed. AFM was first used to compare the surface topography of cells treated with duramycin as a function of exposure time (**Figure 4** and Supplementary Figure S4). Images of the duramycin resistant *Arthrobacter CF158* after treatment with 1 μM duramycin for 1–3 h are shown in **Figure 4**. These images of the resistant strain show healthy cells with a smooth surface at the 1 and 2 h marks. After 3 h treatment, a fraction of the imaged cells (17%), show a spherical morphology and deformation of the cell surface (Supplementary Figure S4). The majority of the cells maintain a rod-shaped morphology. Similarly, *Pseudomonas* GM17 shows no apparent changes in surface roughness or morphology. Images of the duramycin sensitive strains (*Bacillus BC15* and *B. subtilis 168*) before prolonged exposure to duramycin resemble those of *Arthrobacter CF158* in that the cells appear healthy, rod shaped and smooth (**Figure 4**). A wrinkled, spherical cell morphology is observed after just 1 h of treatment with duramycin. With continued duramycin exposure, the cells appear increasingly damaged and after 3 h, most cells appear spherical (80%), with cellular debris surrounding the cell, while the remaining 20% of the cells show severe damage (as shown in **Figure 4** and Supplementary Figure S4).

These high resolution images reveal insights into the mode of action of duramycin. The wrinkled spherical cell shape is characteristic of L-form morphology, where the cell wall peptidoglycan is removed (Errington, 2013). This suggests that exposure to duramycin leads to disruption of the cell wall structure that is the interface between the environment and the cell membrane in the Gram-positive bacteria. The binding of duramycin to the PE head groups likely alters the charge distribution across the membrane and ultimately the cell morphology (Epand et al., 2007). Without the structural support provided by the cell wall, the cells may become fragile and more easily lysed. The role of duramycin in altering cell wall structure is supported further by images of the duramycin conditioned, second-generation *Bacillus BC15* strains (**Figure 4B**). These cells lack PE, based on lipidomics experiments, and display a similar, L-form morphology without duramycin treatment. Finally, the Gram-negative *Pseudomonas* GM17 strain appears unaffected with its membrane encased cell wall.

Cell wall stiffness measurements further support the observed chemical, structural, and morphological changes. Force distance curves were obtained by force volume mapping the surfaces of the four different bacterial species. The results are shown in Supplementary Figure S5 where the X-axis records Young’s modulus and the Y-axis determines the elasticity distribution. Elasticity measurements were collected before and after 3 h treatment with 1 μM duramycin. The sensitive strains show a decreased Young’s modulus after duramycin exposure while the duramycin resistant strains retain their elasticity. The observed decrease in cell wall elasticity is consistent with L-form morphology and increased cellular fragility (Doktycz et al., 2003; Errington, 2013).

### Adhesion Force Measurement with Duramycin Functionalized AFM Tips

**FIGURE 5:**
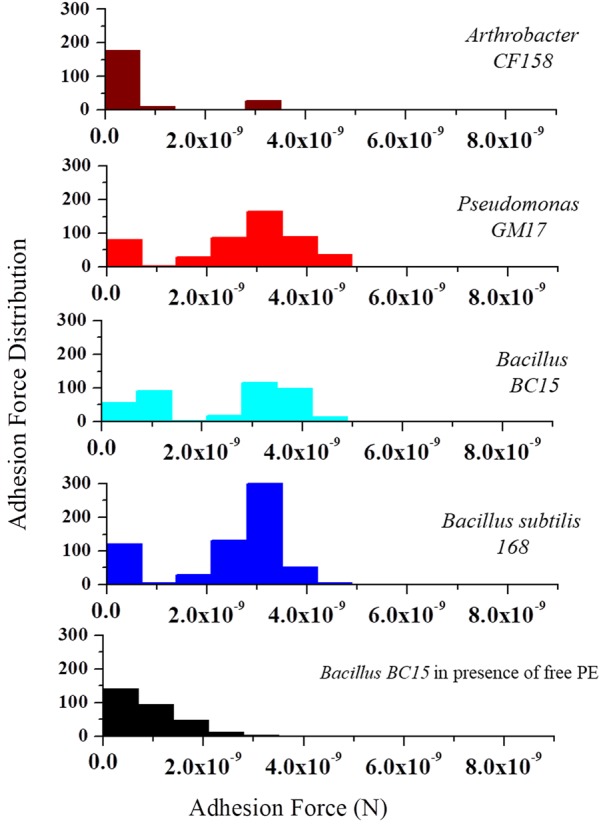
Molecular recognition experiments using duramycin functionalized AFM tips. AFM tips were functionalized with duramycin for interacting with PE on the cell membrane. A graph of the adhesion force frequency versus rupture strength was generated by a 16 × 16 point scan of a 0.5 μm × 0.5 μm sized area on the top of the bacterial cell. An average of 10 different cells from *Arthrobacter CF158, Pseudomonas GM17, Bacillus BC15*, and *B. subtilis 168* were taken. Force curves collected on *Pseudomonas GM17, Bacillus BC15*, and *B. subtilis 168* indicate a high-frequency of strong adhesion between the cell surface and the tip with an adhesion force of ∼3 × 10^-9^ N for the three bacteria. There is also a small amount of adhesion force measured for *Arthrobacter CF158*. In order to show that the interaction of duramycin on the tip is specific for PE, free PE was added to the imaging solution and then the adhesion force was measured using *Bacillus BC15*. This treatment blocks the duramycin functionalized tip and prevents adhesion to PE on the cell surface.

To further analyze the frequency and distribution of PE on the membrane, we used AFM tips functionalized with duramycin to probe the cell surfaces. **Figure 5** represents the histogram of the number of adhesion events occurring between duramycin functionalized tips and the cell surface over a 0.5 μm × 0.5 μm area. These data clearly show common adhesion force values, with a rupture force of 3.5 × 10^-9^ N at a pulling speed of 2.0 μm/s, for those cells identified as containing PE (*B. subtilis 168*, *Bacillus BC15*, and *Pseudomonas GM17*). Under the same conditions, there is also a weaker adhesion force that is observed at (∼1 × 10^-9^ N) that is most frequent for the duramycin resistant *Arthrobacter CF158* strain. The interaction frequency is roughly four times less than that observed for *Pseudomonas GM17*, *Bacillus BC15*, and *B. subtilis 168* and is likely due to non-specific interactions with cell membrane or cell wall. This weak interaction force is also observed in control experiments where PE is added to the imaging solution in order to block the PE binding site of the tethered duramycin. The measured adhesion force between the sensitive duramycin conditioned, second-generation cell strains and the duramycin functionalized tip was similar to that of the *Arthrobacter CF158* strain. No binding was observed at 3.5 × 10^-9^ N for these cells (Supplementary Figure S6). These single molecule adhesion force measurements confirm the determinations obtained from lipidomic studies and, further, establish the location of PE on the outer membrane leaflet for the duramycin sensitive and Gram-negative cells.

## Conclusion

Duramycin inhibition assays on natural bacterial isolates collected from the roots of *Populus* trees show that diverse bacterial strains are sensitive to the lantibiotic. The *Populus* isolate *Bacillus BC15* and the known susceptible strain *B. subtilis 168* are highly sensitive in contrast to the highly resistant *Arthrobacter CF158* and *Pseudomonas GM17*, which showed inhibition only when duramycin concentration was increased by 2–3 orders of magnitude. Imaging with labeled duramycin and mass spectrometry based lipidomics support antibiotic activity screening tests by showing increased binding in strains with higher PE content. Exposure of these sensitive strains to duramycin is not bacteriocidal, as the majority of cells remain viable after 6 h of duramycin exposure. Furthermore, exposure to duramycin leads to development of resistance through decreased incorporation of PE into the cell membrane. In contrast, the largely resistant, *Pseudomonas* GM17 strain contains relatively large amounts of PE even in cells that have been exposed to duramycin. However, the double membrane structure and encapsulated peptidoglycan of this Gram-negative organism likely reduce sensitivity to duramycin. For all four strains, exposure to duramycin leads to clear changes in membrane lipid composition.

High resolution imaging by atomic force microscopy shows that duramycin disrupts cell surface structure and cell morphology. A clear progression of cell surface roughening, morphological changes and cellular destruction are observed when comparing time course images of the sensitive strains against those of the resistant strains. These data are consistent with quantitative measurements of cell membrane elasticity. Collectively, the high-resolution imaging and elasticity data indicate that duramycin binding modifies cell wall organization and structure in Gram-positive bacteria. This may result from disruption of charge pairing between the cell wall constituents and/or reorganization of the membrane lipid components to compensate for the altered charge density. The net result is degeneration of the cell wall, causing increased fragility and reshaping of the rod shaped cell (Muchova et al., 2011). After conditioning with duramycin, subsequent generations of these same bacteria cease expression of PE and maintain this L-form like morphology. The resistant, Gram-negative *Pseudomonas GM17* strain with its membrane protected cell wall does not show altered morphology.

Using duramycin functionalized cantilevers, the single-molecule adhesion force measurements further support the presence and accessibility of PE. Two distinct interaction forces are observed. The weaker interaction is present in all tested strains and likely originates from non-specific interactions. This weaker, non-specific interaction may serve to attract and concentrate the antimicrobial peptide to the surface. The stronger binding force correlated with strains containing PE in their membrane and was eliminated when extraneous PE was added to the imaging solution. This specific PE binding interaction further confirms the presence of PE and its location in the outer leaflet of the membrane.

The complementary biochemical and physical characterizations presented here reveal new details of the selectivity and mode of action of duramycin. The described cellular and molecular measurements can be extended to the study of other complex natural products and aid in the quest of targeting microbial pathogens or the reshaping of microbiome membership.

## Author Contributions

SH, DA, BM, AF, DP, SR, SC, TR, and MD contributed to the design of the study. All authors contributed to the acquisition and analysis of the data and the writing of the manuscript.

## Conflict of Interest Statement

The authors declare that the research was conducted in the absence of any commercial or financial relationships that could be construed as a potential conflict of interest.
