# Supplementary material for: Elucidating Duramycin’s Bacterial Selectivity and Mode of Action on the Bacterial Cell Envelope
Source: Front Microbiol. 2018 Feb 14;9:219. doi: 10.3389/fmicb.2018.00219 (PMC5817074; doi:10.3389/fmicb.2018.00219)
Supplement: Supplementary file 1 [file Data_Sheet_1.DOCX]

**Supporting Information**

**Elucidating duramycin’s bacterial selectivity and mode of action on the bacterial cell envelope**

Sahar Hasim^1,2^, David P. Allison^2, 3^, Berlin Mendez^2^, Abigail T. Farmer^4^, Dale A. Pelletier^2^, Scott T. Retterer^2, 5^, Shawn R. Campagna^4^, Todd B. Reynolds^1^, Mitchel J. Doktycz^2, 5,*^

^1^Dept. Microbiology, University of Tennessee, Knoxville, Tennessee, USA. ^2^Biosciences Division, Oak Ridge National Laboratory, Oak Ridge, Tennessee, USA. ^3^Dept. Biochemistry & Cellular & Molecular Biology, University of Tennessee, Knoxville, Tennessee, USA, ^4^Department of Chemistry, University of Tennessee, Knoxville, Tennessee, USA, and ^5^Center for Nanophase Materials Sciences, Oak Ridge National Laboratory, Oak Ridge, Tennessee, USA.

*Corresponding author, [doktyczmj@ornl.gov](mailto:doktyczmj@ornl.gov)


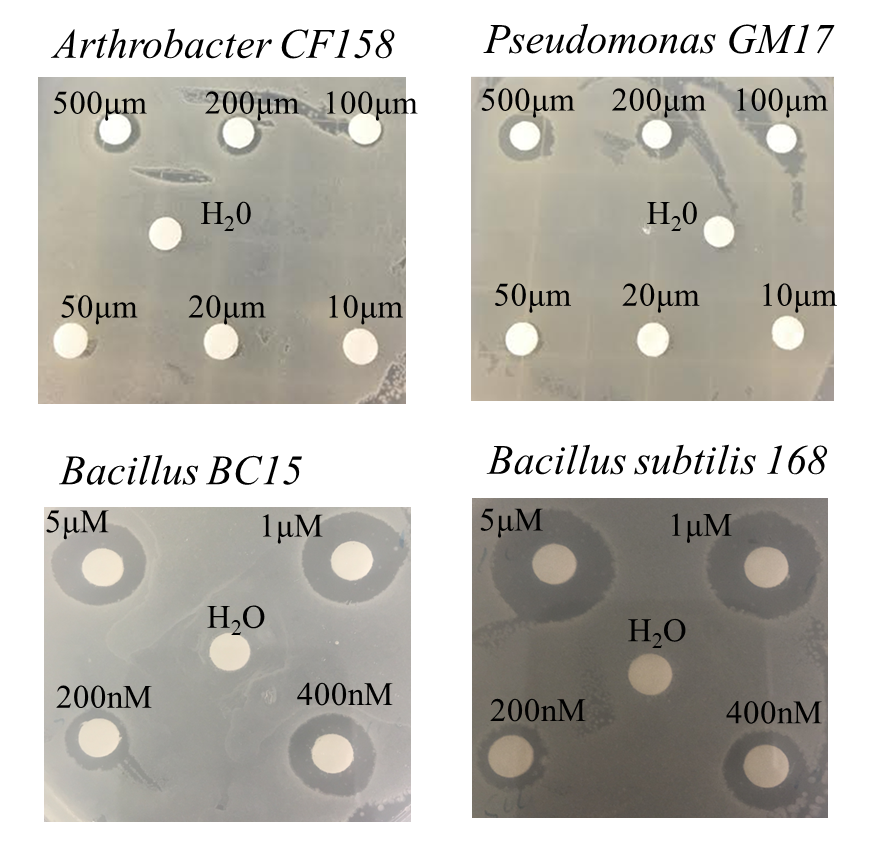


**Figure S1.** **Bacterial sensitivity to duramycin on R2A agar.** Two µL of different concentrations of duramycin were spotted onto small circular filter paper disks on the center of each bacterial lawn and allowed to grow overnight. Shown are the four strains selected for further study including two sensitive Gram-positive strains, *Bacillus BC15* and *Bacillus subtilis* *168*, and the resistant Gram-positive *Arthrobacter CF158* and resistant Gram-negative *Pseudomonas GM17.* The bacterial lawns of the resistant *Arthrobacter CF158* and *Pseudomonas GM17* strains show smaller clearance zones than the sensitive *Bacillus BC15* and *Bacillus subtilis 168* strains.

**
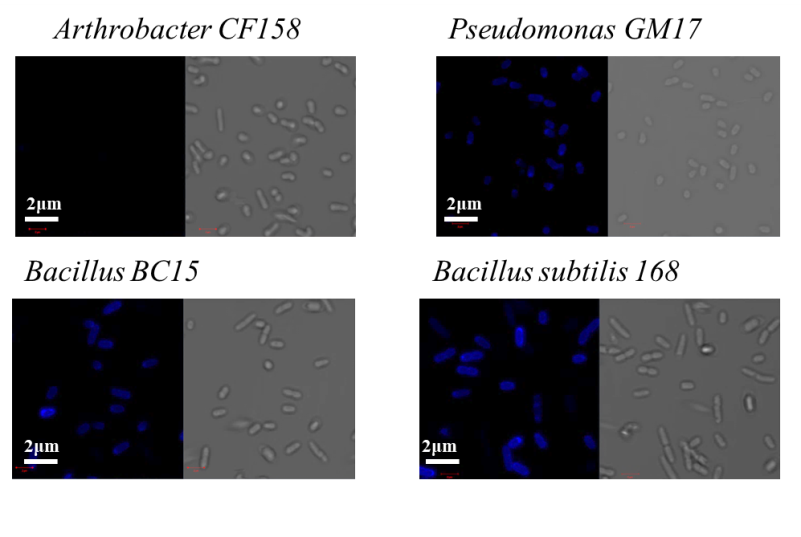
**

**Figure S2) Duramycin-LC-fluorescein staining**

Fluorescent and DIC images of *Arthrobacter* *CF158* and *Pseudomonas GM17, Bacillus BC15* and *Bacillus subtilis 168* with duramycin-LC-fluorescein. Duramycin-LC-fluorescein was used to detect PE on the membrane of each strain. The cells were co- incubated with duramycin-LC-fluorescein for 45 min prior to observation under a confocal microscope. Except for *Arthrobacter CF158* the rest of the strains show localization of the dye to PE on the membrane.


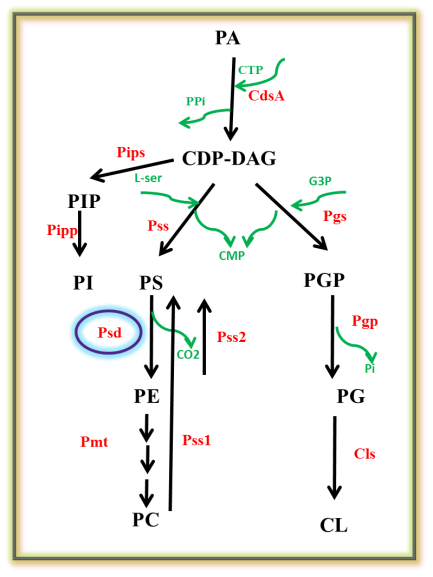


**Figure S3) Phospholipid Biosynthesis Pathway in Bacteria** Overview of phopholipid biosynthetic pathways in bacteria (adapted from Aktas et.al)^1^. PE is produced via decarboxylation of PS by the action of PSD. Sensitive bacteria appear to form PE using the PSD gene product in the pathway whereas the resistance bacteria use alternative pathways (PI and PG).

PA, Phosphatidic acid; CMP, cytidine monophosphate; PI, inositol 1-phosphate;Pipp, PIP phosphatase; CTP, cytidine triphosphate; G3P, glycerol 3-phosphatelys; L-ser, L-serine;CDP-; DAG synthase (CdsA); PS synthases (Pss); PG synthases (Pgs); PS decarboxylases (Psd); PGP phosphatases (Pgp;cardiolipin synthase (Cls); phospholipid *N*-methyltransferase (Pmt).

**^1^Aktas M, Narberhaus F.** 2015. Unconventional membrane lipid biosynthesis in Xanthomonas campestris. Environ Microbiol **17:**3116-3124.

**
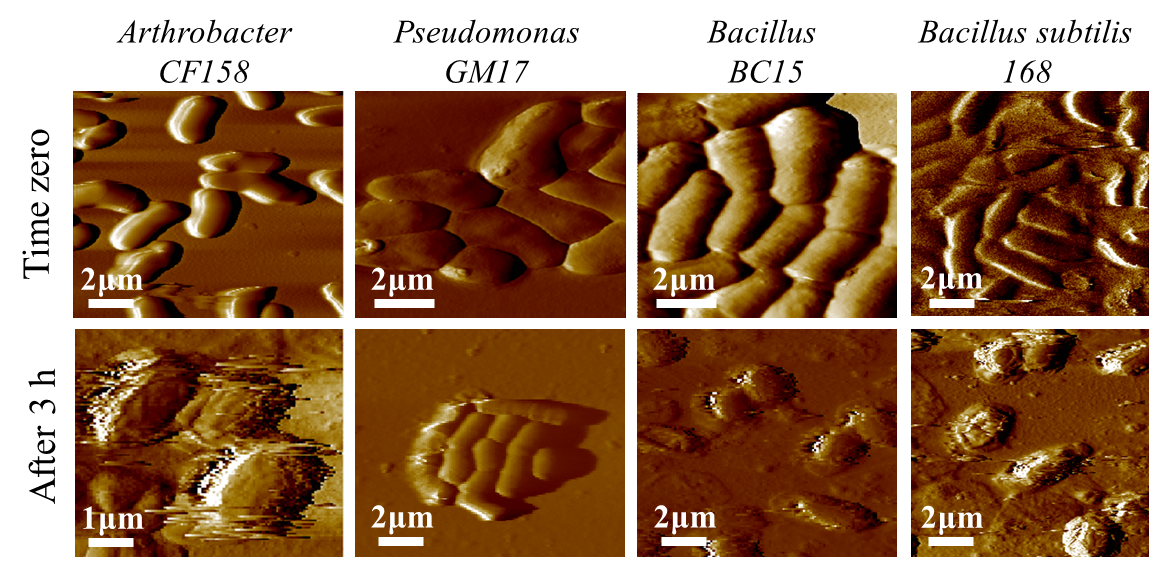
**

**Figure S4)** **Cell morphology of resistant and sensitive strains**

AFM images of cells after 3h treatment with duramycin. The cell morphology of sensitive strains changes to a spherical shape while the morphology of the resistant strains mostly maintains a rod shape.


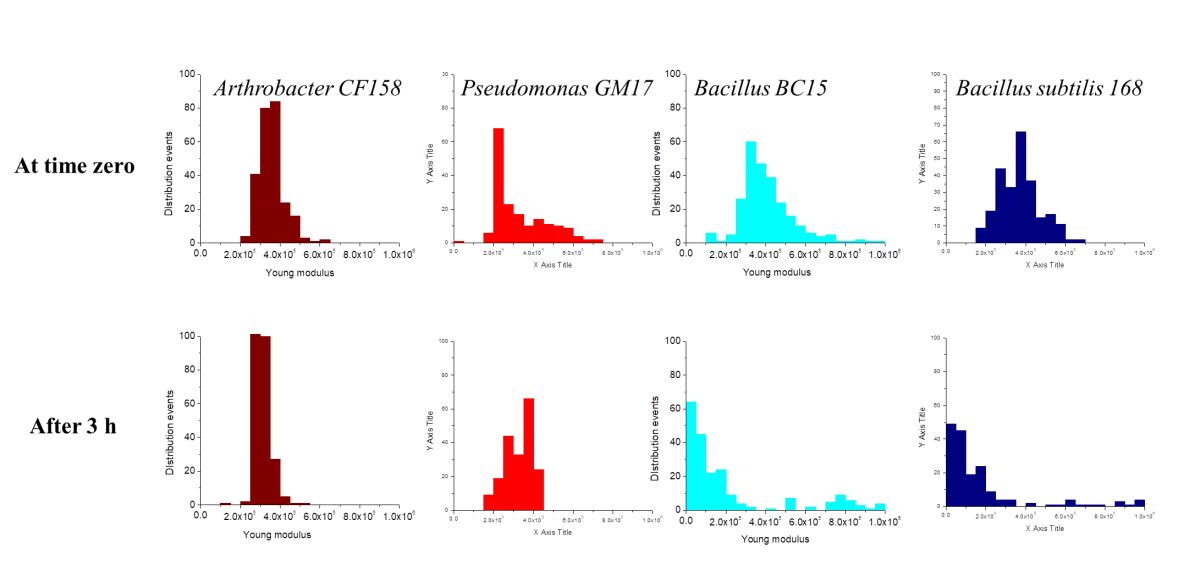


**Figure S5) Elasticity measurements at different time points following duramycin treatment.** Force volume maps of the cell surface were taken by scanning 0.5 x 0.5 μm area on the top of a cell and recording an array of 16-by-16 points, with each point being the average of 3 force curves. The percent distributions of Young's modulus values corresponding to the elasticity of the sensitive and resistant strains were measured at time zero and after three hours treatment with duramycin. The elasticity of sensitive strains increase (decrease in Young’s modulus) after treatment with duramycin. These indentation data were converted into elasticity using the PicoPlus AFM software.

**
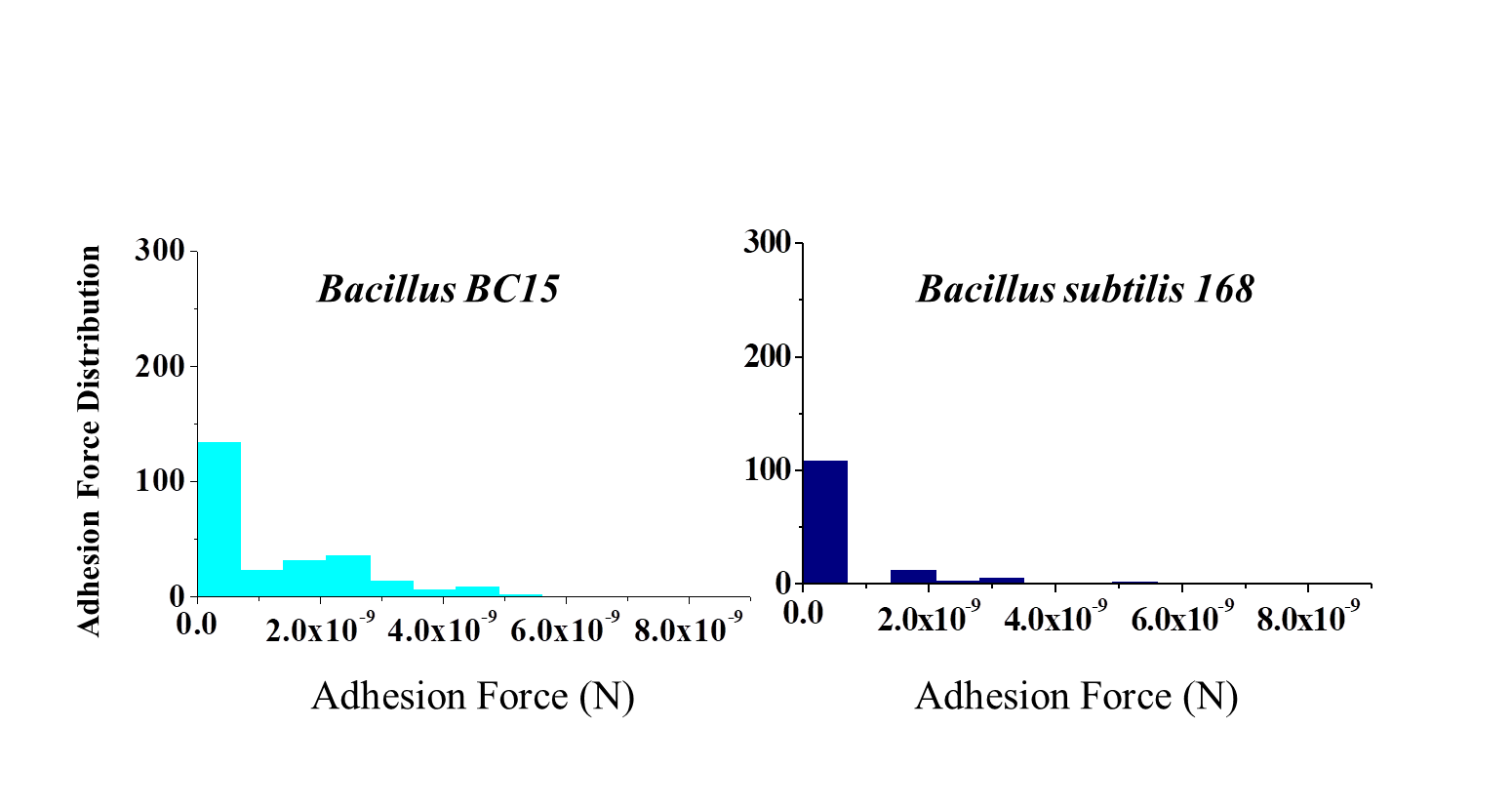
**

**Figure S6) Measurement of second generation adhesion force** Adhesion force volume maps using duramycin functionalized cantilevers recorded on second generation *Bacillus BC15 and Bacillus subtilis 168.* The histogram data shows that there is primarily weak, nonspecific interaction observed between the duramycin-coated cantilever tip and the surface of the second generation cells.

**Table S1. Colony counting after 3&6 hours growth on R2A agar plate with and without duramycin**

**
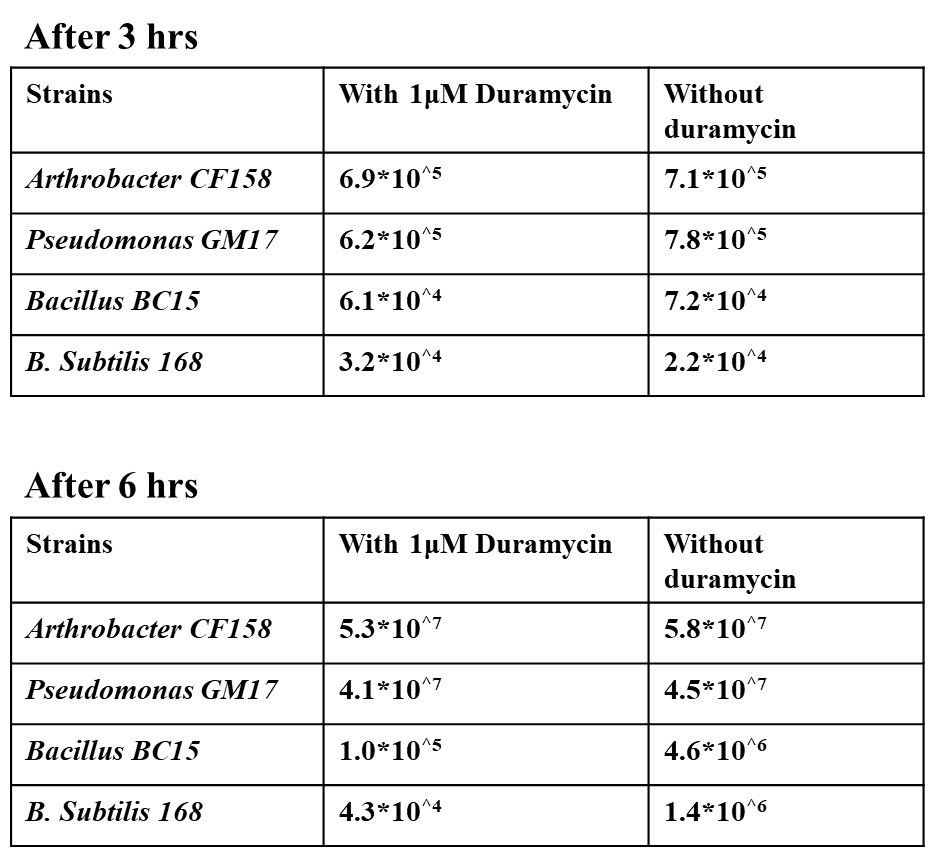
**

S1. To show that the growth inhibition by duramycin is bacteriostatic and not bactericidal, bacterial colony counts after 3 hours and 6 hours of growth in R2A media with and without duramycin were collected. In each case, 100 μL of culture was diluted 3 times and plated on R2A media without duramycin. The viable cells were counted after overnight incubation at 30°C.

**Table S2. First generation lipidomics**

| First generation  Phospholipids composition |  | *Arthrobacter CF158*  *( peak area)* | *Pseudomonas GM17*  *( peak area)* | *Bacillus BC15*  *( peak area)* | *B. subtilis*  *168*  *( peak area)* |
| --- | --- | --- | --- | --- | --- |
| Phosphatidylserine  (PS) | PS(34:2) | 0.00 | 393969.88 | 0.00 | 0.00 |
|  | PS(32:1) | 0.00 | 400608.36 | 0.00 | 2922.85 |
| Phosphatidylethanolamine (PE) | PE(26:0) | 110538.93 | 77530.49 | 1224518.23 | 180946.44 |
|  | PE(28:2) | 4488.86 | 94612.81 | 235892.32 | 0.00 |
|  | PE(28:1) | 3169.30 | 268425.96 | 5605500.49 | 235378.56 |
|  | PE(28:0) | 464786.82 | 253668.25 | 17406086.05 | 1325973.48 |
|  | PE(30:2) | 2160.52 | 169997.14 | 2296878.84 | 4598.86 |
|  | PE(30:1) | 15506.17 | 1709403.23 | 12979725.03 | 411491.58 |
|  | PE(30:0) | 78895.97 | 1608657.25 | 17775201.18 | 50261480.36 |
|  | PE(32:3) | 0.00 | 48935.40 | 504418.70 | 4089.39 |
|  | PE(32:2) | 191509.73 | 4285050.43 | 3709305.98 | 105779.36 |
|  | PE(32:1) | 251026.36 | 61887976.84 | 15513722.53 | 5307518.79 |
|  | PE(32:0) | 46242.73 | 3747622.45 | 6660937.01 | 27353187.46 |
|  | PE(34:2) | 71533.65 | 25183111.58 | 333806.25 | 18314.74 |
|  | PE(34:1) | 57103.72 | 5016768.86 | 853794.18 | 18659.32 |
|  | PE(36:2) | 9465.44 | 231470.40 | 5882.60 | 6042.77 |
|  | PE(40:6) | 0.00 | 3117417.45 | 193777.90 | 70065.56 |
|  | PE(42:7) | 872.04 | 1207261.19 | 0.00 | 0.00 |
|  | PE(26:0) | 0.00 | 484311.64 | 290956.48 | 2953.06 |
| Lyso Phosphatidylethanolamine  (Lyso PE) | lysoPE(14:0) | 0.00 | 23819017.70 | 474644.31 | 3571.24 |
|  | lysoPE(16:1) | 5691.25 | 181338578.10 | 482523.87 | 395653.52 |
|  | lysoPE(16:0) | 0.00 | 109308654.80 | 6427.36 | 3092.33 |
|  | lysoPE(18:1) | 0.00 | 0.00 | 0.00 | 0.00 |
| Phosphatidic acid (PA) | PA(36:1) | 2395381.86 | 3539.49 | 421199.27 | 3962596.38 |
|  | PA(30:0) | 1782.07 | 1049593.66 | 290652.18 | 621270.49 |
|  | PA(32:1) | 770604.90 | 110243.85 | 291804.30 | 4074081.02 |
|  | PA(32:0) | 320.68 | 674229.19 | 11971.10 | 2315.01 |
|  | PA(34:2) | 3904.89 | 124080.25 | 14703.02 | 7200.31 |
|  | PA(34:1) | 14259.86 | 1097.12 | 36093.41 | 25052.05 |
|  | PA(34:0) | 0.00 | 0.00 | 24537.21 | 0.00 |
| Phosphatidylglycerol  (PG) | PG(36:0) | 144626.82 | 76202.74 | 398695.15 | 55340.73 |
|  | PG(26:0) | 26965.05 | 63496.52 | 2434262.21 | 912.45 |
|  | PG(28:1) | 1745247.79 | 104715.09 | 7468791.14 | 459318.79 |
|  | PG(28:0) | 5467.64 | 186168.98 | 3015090.95 | 0.00 |
|  | PG(30:2) | 3630237.15 | 931194.17 | 17474943.64 | 227908.69 |
|  | PG(30:1) | 142592196.80 | 1146891.27 | 15871690.58 | 21789953.34 |
|  | PG(30:0) | 3490.81 | 39056.96 | 905066.08 | 1573.50 |
|  | PG(32:3) | 12441.40 | 13339763.45 | 6009941.99 | 6331.10 |
|  | PG(32:2) | 149607.08 | 126277898.90 | 31217570.53 | 7764748.30 |
|  | PG(32:1) | 3685832.09 | 10228323.80 | 16716794.13 | 51239945.36 |
|  | PG(32:0) | 58842.17 | 53329455.85 | 1291266.03 | 11723.55 |
|  | PG(34:2) | 4243.90 | 0.00 | 9933.59 | 0.00 |
| Phosphatidylinositol  (PI) | PI(34:1) | 296722.11 | 0.00 | 3795.15 | 46542.29 |
|  | PI(36:2) | 735708.72 | 453.90 | 106944.38 | 253749.47 |
|  | PI(28:1) | 421786.26 | 0.00 | 0.00 | 2637.33 |
|  | PI(28:0) | 42598163.69 | 86364.79 | 35072.39 | 2056.82 |
|  | PI(30:0) | 1962514.05 | 0.00 | 0.00 | 0.00 |
|  | PI(32:0) | 236403.75 | 0.00 | 21557.66 | 237796.15 |
|  | PI(38:2) | 79819.96 | 286732.50 | 1204994.12 | 358813.83 |
| Cardiolipin  (CL) | CL(64:0) | 2127.31 | 630828.20 | 476892.64 | 27068.74 |
|  | CL(72:5) | 2120.13 | 529210.33 | 296794.16 | 101786.48 |
|  | CL(72:6) | 345456.94 | 897234.54 | 1001090.87 | 3650664.93 |
|  | CL(70:3) | 29227.23 | 16907.73 | 1847043.51 | 701834.65 |
|  | CL(70:4) | 75974.25 | 114392.21 | 1725135.90 | 149785.49 |
|  | CL(70:5) | 112714.71 | 23418.00 | 689021.13 | 13730.78 |
|  | CL(70:6) | 1479392.09 | 2330344.56 | 418966.90 | 681485.24 |
|  | CL(68:2) | 3465516.90 | 136535.52 | 1554851.60 | 1923284.15 |
|  | CL(68:3) | 91774.60 | 424605.03 | 1950141.98 | 285229.40 |
|  | CL(68:4) | 19521.30 | 8153.80 | 1312009.59 | 7027.99 |
|  | CL(68:5) | 41632.83 | 5232849.86 | 509963.66 | 37139.88 |
|  | CL(66:1) | 5303547.63 | 1229462.23 | 982114.73 | 212768.35 |
|  | CL(66:3) | 73718.26 | 265962.29 | 1170310.74 | 40947.03 |
|  | CL(66:4) | 247724.17 | 1610062.00 | 272035.03 | 290063.64 |
|  | CL(64:2) | 221970.21 | 88428.54 | 1231909.60 | 1963234.53 |
|  | CL(62:0) | 45730.54 | 81550.61 | 546152.60 | 687793.71 |
|  | CL(62:1) | 5962812.15 | 179156.66 | 1045335.38 | 1136275.82 |
|  | CL(60:0) | 117694.02 | 20495.83 | 654998.80 | 129557.05 |
|  | CL(60:1) | 431756.04 | 4281.73 | 150239.90 | 101785.45 |
|  | CL(58:0) | 0.00 | 0.00 | 7724.67 | 249489.31 |
| IPC | IPC(32:2) | 220817.16 | 329086.09 | 458353.05 | 199102.38 |
|  | PC(28:6) | 399436.49 | 200627.00 | 2921574.32 | 304500.71 |
| Phosphatidylcholine  (PC) | PC(28:4) | 0.00 | 7843063.62 | 18144018.53 | 1212689.05 |
|  | PC(30:1) | 53863704.10 | 38567175.76 | 80016437.72 | 25695264.93 |
|  | PC(32:6) | 1573950.98 | 1220404.67 | 2249119.95 | 774944.28 |
|  | PC(32:5) | 0.00 | 149662094.90 | 0.00 | 0.00 |
|  | PC(32:1) | 330284.06 | 40048635.16 | 0.00 | 0.00 |
|  | PC(34:2) | 0.00 | 9581433.18 | 0.00 | 0.00 |
|  | PC(34:1) | 312420.66 | 304164.62 | 8079.23 | 69023.83 |
|  | PC(36:2) | 0.00 | 0.00 | 401.95 | 0.00 |
|  | PC(36:0) | 416861.79 | 546509.49 | 444161.48 | 520835.48 |
|  | PC(38:2) |  | 393969.88 | 0.00 |  |

**Table S3. Second generation lipidomics**

| Second generation  Phospholipids composition |  | *Arthrobacter CF158*  *( peak area)* | *Pseudomonas GM17*  *( peak area)* | *Bacillus BC15*  *( peak area)* | *B. subtilis*  *168*  *( peak area)* |
| --- | --- | --- | --- | --- | --- |
| Phosphatidylserine  (PS) | PS(34:2) | 3890.00 | 1675000.00 | 4790000.00 | 0.00 |
|  | PS(26:0) | 0.00 | 0.00 | 1016500.00 | 0.00 |
|  | PS(28:2) | 0.00 | 0.00 | 1038500.00 | 0.00 |
|  | PS(28:1) | 0.00 | 0.00 | 17100000.00 | 0.00 |
|  | PS(28:0) | 0.00 | 0.00 | 52150000.00 | 1290000.00 |
|  | PS(30:3) | 0.00 | 0.00 | 519000.00 | 0.00 |
|  | PS(30:2) | 0.00 | 0.00 | 24550000.00 | 0.00 |
|  | PS(30:1) | 0.00 | 21250.00 | 184000000.00 | 5400.00 |
|  | PS(30:0) | 6390.00 | 106950.00 | 140000000.00 | 11600000.00 |
|  | PS(32:3) | 0.00 | 0.00 | 6560000.00 | 4780.00 |
|  | PS(32:2) | 0.00 | 159500.00 | 54900000.00 | 0.00 |
|  | PS(32:1) | 7780.00 | 11550000.00 | 252000000.00 | 591000.00 |
|  | PS(32:0) | 3010.00 | 529500.00 | 110600000.00 | 170000000.00 |
|  | PS(34:4) | 0.00 | 8390.00 | 1115000.00 | 0.00 |
|  | PS(34:1) | 2450.00 | 756500.00 | 7820000.00 | 20000.00 |
|  | PS(34:0) | 8380.00 | 47250.00 | 4185000.00 | 10600.00 |
| Phosphatidylethanolamine (PE) | PE(26:0) | 206000.00 | 2198659.50 | 274.57 | 62200.00 |
|  | PE(26:1) | 7630.00 | 916700.15 | 10536.08 | 0.00 |
|  | PE(28:0) | 160000.00 | 9170918.50 | 30445.00 | 971000.00 |
|  | PE(28:1) | 2810.00 | 8084533.50 | 70132.50 | 2700.00 |
|  | PE(28:2) | 1970.00 | 1471193.00 | 0.00 | 0.00 |
|  | PE(30:0) | 240000.00 | 82750000.00 | 493527.50 | 451000.00 |
|  | PE(30:1) | 51500.00 | 66150000.00 | 1325000.00 | 33500.00 |
|  | PE(30:2) | 6180.00 | 4813816.00 | 188093.00 | 0.00 |
|  | PE(32:0) | 83100.00 | 421500000.00 | 763753.50 | 227000.00 |
|  | PE(32:1) | 1690.00 | 4100000000.00 | 4225000.00 | 983000.00 |
|  | PE(32:2) | 72800.00 | 139500000.00 | 568623.00 | 107000.00 |
|  | PE(32:3) | 4660.00 | 525828.15 | 23994.70 | 8160.00 |
|  | PE(34:0) | 2210.00 | 34150000.00 | 8983.83 | 419.00 |
|  | PE(34:2) | 284000.00 | 1590000000.00 | 858278.00 | 311000.00 |
|  | PE(34:3) | 0.00 | 2550905.50 | 0.00 | 0.00 |
|  | PE(36:2) | 1320.00 | 29450000.00 | 5733.67 | 0.00 |
|  | PE(40:6) | 2160.00 | 50850000.00 | 247730.00 | 13100.00 |
|  | PE(40:7) | 0.00 | 3613559.50 | 323.58 | 0.00 |
|  | PE(40:8) | 0.00 | 0.00 | 0.00 | 0.00 |
|  | PE(42:7) | 2170.00 | 23000000.00 | 0.00 | 11800.00 |
| Lyso Phosphatidylethanolamine  (Lyso PE) | lysoPE(14:0) | 6200.00 | 46264760.00 | 0.00 | 0.00 |
|  | lysoPE(16:0) | 0.00 | 7125000000.00 | 129436.45 | 15700.00 |
|  | lysoPE(16:1) | 22900.00 | 240000000.00 | 10542.25 | 5420.00 |
|  | lysoPE(18:1) | 0.00 | 480000000.00 | 59178.64 | 17100.00 |
| Phosphatidic acid (PA) | PA(30:0) | 633000000.00 | 76199.17 | 2128066.50 | 19800000.00 |
|  | PA(32:0) | 90300000.00 | 204829.95 | 4607621.00 | 24800000.00 |
|  | PA(32:1) | 28600.00 | 10050195.50 | 2680173.00 | 296000.00 |
|  | PA(34:0) | 1720000.00 | 3205.49 | 271865.90 | 120000.00 |
|  | PA(34:2) | 13700.00 | 3965527.50 | 19018.44 | 3530.00 |
|  | PA(36:1) | 0.00 | 19839.84 | 9553.49 | 0.00 |
| Phosphatidylglycerol  (PG) | PG(26:0) | 3690000.00 | 186405.85 | 520534.25 | 614000.00 |
|  | PG(28:0) | 123000000.00 | 3179668.00 | 127000000.00 | 20700000.00 |
|  | PG(28:1) | 598000.00 | 2848140.00 | 31500000.00 | 674000.00 |
|  | PG(30:0) | 8760000000.00 | 52750000.00 | 793000000.00 | 855000000.00 |
|  | PG(30:1) | 36000000.00 | 41700000.00 | 414500000.00 | 2720000.00 |
|  | PG(30:2) | 14500.00 | 3777673.50 | 28000000.00 | 1410000.00 |
|  | PG(32:0) | 404000000.00 | 246000000.00 | 1200000000.00 | 255000000.00 |
|  | PG(32:1) | 7170000.00 | 2980000000.00 | 975000000.00 | 38700000.00 |
|  | PG(32:2) | 0.00 | 312500000.00 | 96950000.00 | 786000.00 |
|  | PG(32:3) | 0.00 | 441317.45 | 6214020.00 | 317000.00 |
|  | PG(34:1) | 506000000.00 | 375500000.00 | 70500000.00 | 2120000.00 |
|  | PG(34:2) | 1380000.00 | 1260000000.00 | 19500000.00 | 58500.00 |
|  | PG(36:0) | 15600000.00 | 15068.78 | 775245.00 | 7210000.00 |
|  | PG(36:2) | 1060000.00 | 23750000.00 | 5153860.50 | 486000.00 |
|  | PG(42:10) | 1960000.00 | 4138644.00 | 14600000.00 | 3430000.00 |
|  | PG(42:8) | 0.00 | 9765.96 | 1758459.00 | 511000.00 |
|  | PG(42:9) | 0.00 | 284726.55 | 13100000.00 | 6850000.00 |
|  | PG(44:9) | 0.00 | 0.00 | 2921925.00 | 542000.00 |
| Phosphatidylinositol  (PI) | PI(36:2) | 48200000.00 | 3860.00 | 17200.00 | 23000.00 |
|  | PI(30:0) | 4290000000.00 | 390500.00 | 259500.00 | 60500.00 |
|  | PI(32:0) | 580000000.00 | 6295000.00 | 5455000.00 | 5100000.00 |
|  | PI(38:2) | 5150000.00 | 0.00 | 0.00 | 0.00 |
|  | PI(28:1) | 30400000.00 | 219000.00 | 4105000.00 | 6700000.00 |
|  | PI(28:0) | 6120000.00 | 5000.00 | 22610.00 | 25000.00 |
| Cardiolipin  (CL) | CL(58:0) | 12600000.00 | 29444.58 | 721592.60 | 901000.00 |
|  | CL(62:1) | 212000.00 | 1046564.00 | 9603432.50 | 3040000.00 |
|  | CL(64:2) | 790000.00 | 30250000.00 | 7569691.00 | 3350000.00 |
|  | CL(66:1) | 31700.00 | 181000000.00 | 1090996.20 | 177000.00 |
|  | CL(66:3) | 75400000.00 | 882003.00 | 8160022.00 | 2810000.00 |
|  | CL(66:4) | 29200.00 | 105074.40 | 7145412.50 | 0.00 |
|  | CL(68:2) | 18100.00 | 115500000.00 | 116543.10 | 50700.00 |
|  | CL(68:3) | 307000000.00 | 160678.59 | 25550000.00 | 31700000.00 |
|  | CL(68:4) | 393000.00 | 7788.71 | 26400000.00 | 73100.00 |
|  | CL(68:5) | 0.00 | 7551.63 | 9737826.50 | 0.00 |
|  | CL(70:3) | 183000000.00 | 642622.55 | 35100000.00 | 51000000.00 |
|  | CL(70:4) | 3890.00 | 1675000.00 | 4790000.00 | 0.00 |
|  | CL(70:5) | 0.00 | 0.00 | 1016500.00 | 0.00 |
|  | CL(70:6) | 0.00 | 0.00 | 1038500.00 | 0.00 |
|  | CL(72:5) | 0.00 | 0.00 | 17100000.00 | 0.00 |
|  | CL(72:6) | 0.00 | 0.00 | 52150000.00 | 1290000.00 |
| Phosphatidylcholine  (PC) | PC(26:4) | 0.00 | 0.00 | 519000.00 | 0.00 |
|  | PC(36:2) | 0.00 | 0.00 | 24550000.00 | 0.00 |
|  | PC(36:3) | 0.00 | 21250.00 | 184000000.00 | 5400.00 |
|  | PC(36:4) | 6390.00 | 106950.00 | 140000000.00 | 11600000.00 |
|  | PC(34:2) | 0.00 | 0.00 | 6560000.00 | 4780.00 |
|  | PC(34:3) | 0.00 | 159500.00 | 54900000.00 | 0.00 |
|  | PC(32:1) | 7780.00 | 11550000.00 | 252000000.00 | 591000.00 |
|  | PC(38:2) | 3010.00 | 529500.00 | 110600000.00 | 170000000.00 |

The nomenclature for phospholipids is head group (total number of carbons in acyl chains: total number of double bonds in acyl chains)

The numbers in parentheses refer to the combined number of carbons and unsaturated bonds in the fatty acid component of the phospholipid.
